# Expression Patterns and Functional Novelty of Ribonuclease 1 in Herbivorous *Megalobrama amblycephala*

**DOI:** 10.3390/ijms17050786

**Published:** 2016-05-20

**Authors:** Han Liu, Weimin Wang

**Affiliations:** 1 Key Lab of Agricultural Animal Genetics, Breeding and Reproduction of Ministry of Education, Key Lab of Freshwater Animal Breeding, Ministry of Agriculture, College of Fisheries, Huazhong Agricultural University, Wuhan 430070, China; lifegood1986@126.com; 2 Collaborative Innovation Center for Efficient and Health Production of Fisheries in Hunan Province, Changde 41500, China

**Keywords:** RNase1, *Megalobrama amblycephala*, digestive enzyme, ribonuclease activity

## Abstract

Ribonuclease 1 (RNase1) is an important digestive enzyme that has been used to study the molecular evolutionary and plant-feeding adaptation of mammals. However, the expression patterns and potential biological function of RNase1 in herbivorous fish is not known. Here, we identified *RNase1* from five fish species and illuminated the functional diversification and expression of *RNase1* in herbivorous *Megalobrama amblycephala*. The five identified fish *RNase1* genes all have the signature motifs of the RNase A superfamily. No expression of *Ma*-*RNase1* was detected in early developmental stages but a weak expression was detected at 120 and 144 hours post-fertilization (hpf). *Ma*-*RNase1* was only expressed in the liver and heart of one-year-old fish but strongly expressed in the liver, spleen, gut, kidney and testis of two-year-old fish. Moreover, the immunostaining localized RNase1 production to multiple tissues of two-year-old fish. A biological functional analysis of the recombinant protein demonstrated that *M. amblycephala* RNase1 had a relatively strong ribonuclease activity at its optimal pH 6.1, which is consistent with the pH of its intestinal microenvironment. Collectively, these results clearly show that *Ma*-RNase1 protein has ribonuclease activity and the expression patterns of *Ma*-*RNase1* are dramatically different in one year and two-year-old fish, suggesting the functional differentiation during fish growing.

## 1. Introduction

The ribonuclease A (RNase A) superfamily is a vertebrate-specific enzyme family containing numerous structurally similar proteins [1]. In humans, the RNase A family contains eight different members. As secreted proteins, all of them have a signal sequence at the N-terminus and an invariant catalytic triad, which includes the typical consensus motif CKXXNTF, and 6–8 conserved cysteines forming disulfide bridges [1,2]. Previous studies have demonstrated that many members of the vertebrate RNase A superfamily evolved to perform a large number of different functions, including the degradation of dietary RNAs [3,4], innate immunity [5], angiogenesis [6,7], and antibacterial activities [2,8,9].

Ribonuclease 1 (RNase1), an important digestive enzyme, is mainly secreted by the vertebrate pancreas. Expression studies have shown high expression levels of *RNase1* in the pancreases of humans and ruminants [3,10]. It has been reported that the main biological function of RNase1 in ruminants and some primates is enzymatic, digesting the symbiotic bacteria in their foreguts [3,11]. RNase1 also degrades pathogenic RNA to protect organisms from invasive pathogens [9,12]. Previous studies have shown that the duplication of the *RNase1* gene is linked to the herbivorous diet. For instance, duplications of the *RNase1* gene have been found in foregut-fermenting herbivorous leaf monkeys, in which RNase1 contributes to their functional adaptation to a diet of plant materia [4,13]. One of the two duplicated *RNase1* genes in monkeys is specifically functionally suited to a low-pH environment, which is thought to be an adaptation to the acidified environment of the foregut [13]. However, recent studies have shown that *RNase1* gene duplication is not restricted to foregut-fermenting mammals (colobines and ruminants) but has also been documented in rat species [14], squirrels [15], bats [16], Carnivora [17,18].

Recently, several members of the RNase A family have been identified in fish genomes, including those of *Danio rerio* and *Salmo salar*. Gene expression analyses showed that *D. rerio RNase1* (*Dr-RNase1*) and *Dr-RNase2* are mainly expressed in the adult liver and gut, and are weakly expressed in the heart tissues [19]. A functional analysis of recombinant proteins demonstrated that all three *Dr*-RNases have strong antibacterial activity *in vitro* and except for *Dr*-RNase3, weak ribonucleolytic activity. Similarly, recombinant *S. salar* RNase proteins, *Ss*-RNase1 and *Ss*-RNase2, have both angiogenic and bactericidal activities, but have very weak RNA-degrading activity [8]. However, the expression patterns and biological function of RNase1 in herbivorous fish is not known.

*M. amblycephala* is an economically important cyprinid species in freshwater aquaculture in China. Because it is a typical herbivorous fish, consuming little fish meal and fish oil, it is recognized as an ecofriendly and resource-conserving fish. In this study, we identified the *RNase1* gene in the whole genome data of *M. amblycephala*, *D. rerio*, *Cyprinus carpio*, *Ctenopharyngodon idellus*, and *Oryzias latipes*. The mRNA expression levels of *M. amblycephala RNase1* (*Ma-RNase1*) in various early developmental stages, different tissues of one- and two-year-old fish were determined by quantitative real-time polymerase chain reaction (qPCR). Immunohistochemistry and immunofluorescence analyses were performed to localize the RNase1 peptide production. Further, recombinant *Ma*-RNase1 protein was prepared to determine its biological function.

## 2. Results

### 2.1. Protein Alignment

**Figure 1:**
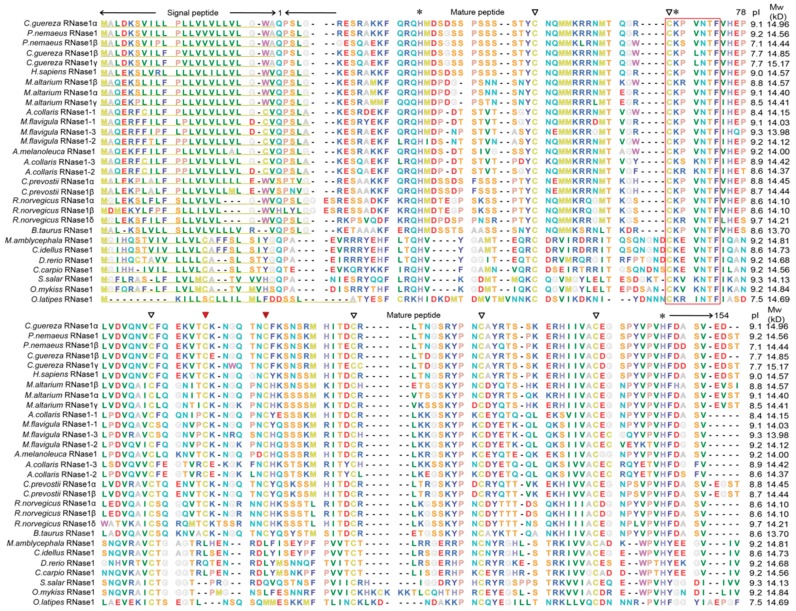
Multiple protein sequences alignment of RNase1 in fishes and mammals. Dashes indicate alignment gaps. Yellow underlines showed the signal peptides. The putative isoelectric point (pI) and molecular weight (*M*w) indicated the isoelectric point and molecular weight (kDa) of the mature peptide, respectively. The conversed CKXXNTF motif is shown in a box. The eight structural cysteines (active-site residues) are marked with triangles and the two red triangles indicated alteration in fishes. Asterisks show the three catalytic resides.

The full-length cDNA of *Ma-RNase1* contains a 453-bp coding sequence, a 107-bp 5′-untranslated region (5′-UTR), and a 94-bp 3′-UTR (Supplementary Figure S1). We performed TBLASTN and BLAST searches of four other fish genomes (*D. rerio*, *C. carpio*, *C. idellus*, and *O. latipes*). Only one putative functional *RNase1* gene was identified in each while previous studies have found multiple copies in mammals [13,14,15,16]. A multiple-protein-sequence alignment showed that all RNase1 proteins have a signal peptide of more than 20 amino acids at the N-terminus (Figure 1). All these sequences have the “CKXXNTF” signature motif, ribonuclease activity required catalytic triad (His12–Lys41–His119, numbers according to the mature peptide of *H. sapiens* RNase1) and conserved cysteine residues. It should be noted that all mammalian RNase1 proteins have eight cysteines, but the five teleosts RNase1 proteins have only six cysteines (Figure 1). The putative isoelectric point (pI) and calculated molecular weight (*M*_W_) of the mature peptide of RNase1 proteins in mammals and teleosts were ranging from 7.1 to 9.7 and 13.70 to 15.17 kD, respectively.

### 2.2. Structural Predictions

**Figure 2:**
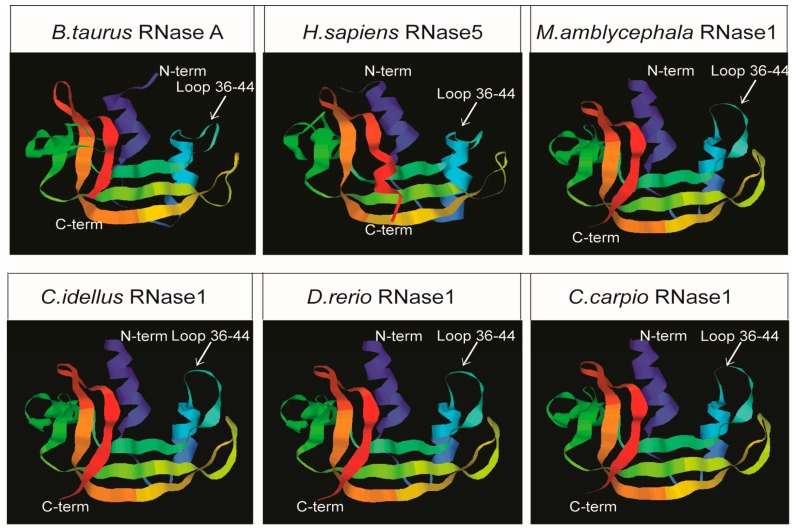
Ribbon representations of 3D structures of four fish RNase1 and two mammals RNase A. The blue, light blue and green ribbons represent the three α-helices and ribbons with others colors represent the six β-strands.

The three-dimensional (3D) structures of *M. amblycephala*, other fishes and mammals RNase1 were predicted by homology modeling (Figure 2). The structure of *Ma*-RNase1 shows a typical fold for members of the RNase A family with three α-helices and six β-strands. As expected from the sequence-structure alignments, the constructed models of *M. amblycephala*, *C. idella*, *D. rerio* and *C. carpio* RNase1 closely resembled each other. Interestingly, the 3D structures of the fish RNase1 were more similar to the structure of *Homo sapiens* RNase5 (angiogenin) than *Bostaurus* RNase A.

### 2.3. Phylogenetic Relationships among the Bony Fish and Mammalian RNase1 Proteins

**Figure 3:**
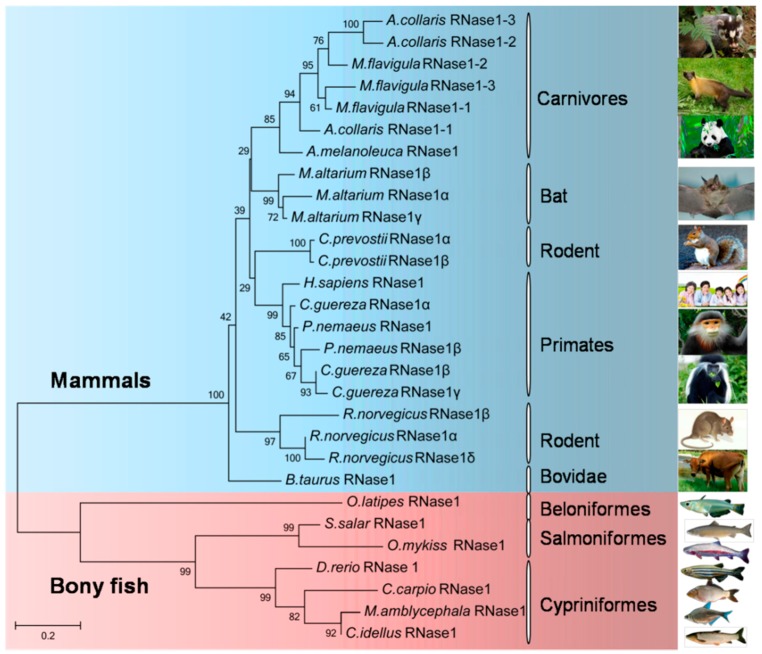
Phylogenetic tree documenting relationships among various vertebrates RNase1. Neighbor-joining tree constructed with protein sequences by MEGA 5.1. Numbers at branches indicate the bootstrap probabilities with 1000 replicates.

To gain a broader picture of the evolution of the vertebrate RNase1 proteins, we reconstructed a protein-based Neighbor-Joining tree for RNase1 in the bony fishes and previously identified mammalian RNase1 proteins. The clade of fish RNase1 was clearly separate from that of mammalian RNase1 (Figure 3). The *Ma*-RNase1 protein sequence is more similar to that of *C. idellus* than to that of *C. carpio* or *D. rerio*. The RNase1 proteins of the four cyprinid fishes were more closely related to each other and clustered in a single clade. It is noteworthy that some mammals, including *Myotis altarium*, *R. norvegicus*, *Callosciurus prevostii*, *Arctonyx collaris, Martes flavigula, P. nemaeus*, and *Colobus guereza*, have multiple copies of the *RNase1* gene, but only a single copy of *RNase1* occurs in the bony fishes. Because the vertebrate RNase1 sequences are short and quite divergent, the bootstrap values on the phylogenetic tree are not high at some nodes.

### 2.4. Ma-RNase1 Expression Patterns

**Figure 4:**
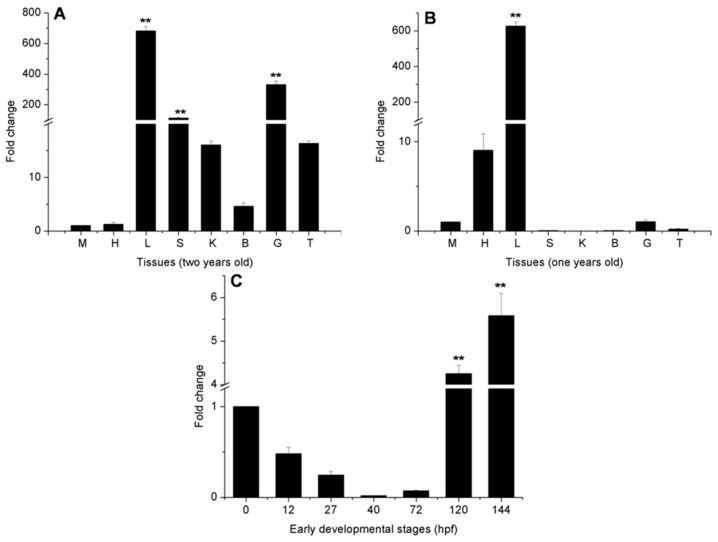
The expression patterns of *RNase1* genes in different developmental stages and tissues of *M. amblycephala*. *B-actin* was used as reference gene. (**A**) Two-year-old fish (the muscle tissue was the same as in the control); (**B**) One-year-old fish (the muscle tissue was the same as in the control); (**C**) early different developmental stages of *M. amblycephala* (0 hpf egg was the same as in the control). cDNAs were used for PCR from total RNA samples obtained from the heart (H), liver (L), spleen (S), kidney (K), brain (B), muscle (M), gut (G) and testis (T) of one year and two-year-old fish, 0 (fertilized egg), 12 (late gastrula stage), 27 (heart appearance), 40 (hatching), 72 (gill circulation), 120 (air bladder formation), 144 (intestine appearance) hours post-fertilization (hpf) embryos/larvae. Differences were determined by one-way analysis of variance (ANOVA). Statistically significant differences from the control group are marked as ** *p* <0.01.

No expression was detected in the early developmental stages (0, 12, 27, 40, or 72 h post-fertilization (hpf)), but weak expression was detected at 120 and 144 hpf (Figure 4). In the one-year-old fish, *Ma-RNase1* was strongly expressed in the liver tissues and weakly expressed in the heart, but no expression was detected in the spleen, kidney, brain, muscle, gut, or testis (Figure 4). Notably, the expression profile of the *Ma-RNase1* gene in the two-year-old fish was dramatically different from that in the one-year-old fish. *Ma-RNase1* expressed in most of the determined tissues in the two-year-old fish. It showed strong expression in the liver, spleen, gut, kidney and testis, relatively weak expression in the brain, and no expression in the heart or muscle tissues.

### 2.5. Ma-RNase1 Peptide Production Analyzed by Immunohistochemistry

**Figure 5:**
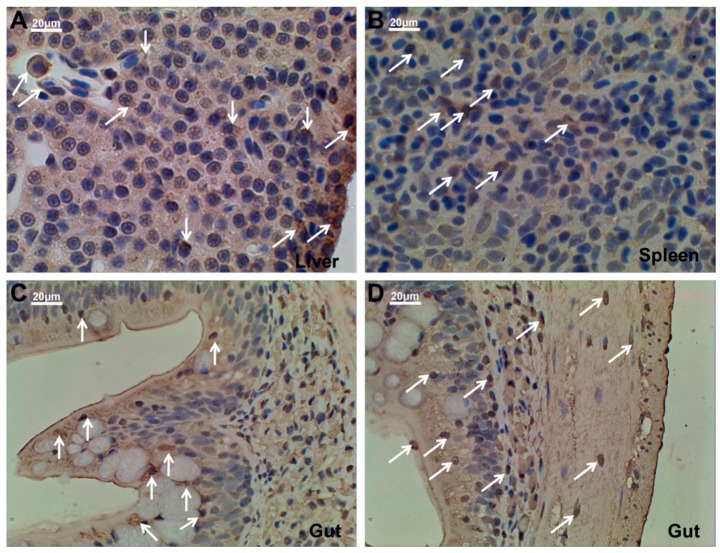
Expression of *Ma*-RNase1 in different tissues of *M. amblycephala*. Immunohistochemistry demonstrates *Ma*-RNase1 production (positive cells labeled in brown showing as white arrows) in the liver (**A**), spleen (**B**) and gut (**C**,**D**) tissues of two-year-old *M. amblycephala*. The negative cell nucleus labeled in blue and positive nucleus labeled in brown.

**Figure 6:**
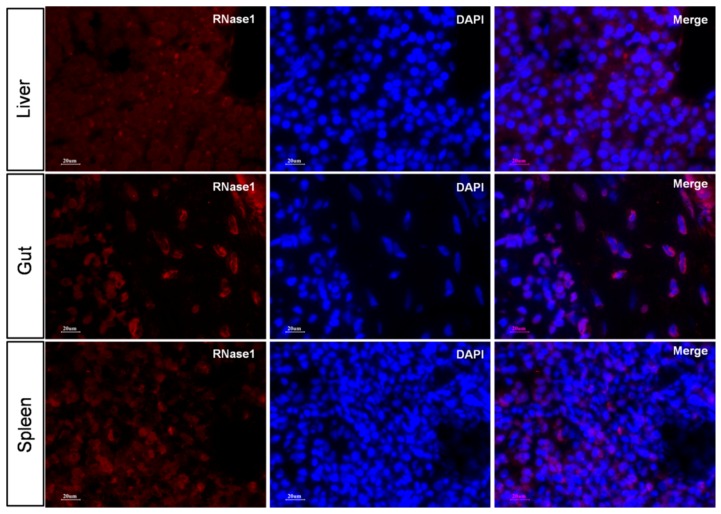
Immunofluorescence analysis demonstrates RNase1 production in the liver, gut and spleen tissues of two-year-old *M. amblycephala*. Immunofluorescence staining identifying cells with RNase1 expression in red and nuclei in blue.

To localize the RNase1 peptide production in different tissues of two-year-old *M. amblycephala*, we performed immunohistochemistry analysis using polyclonal antibodies directed against RNase1. The results clearly showed that RNase1 was best expressed throughout the liver and gut tissues, and relatively weakly expressed in spleen (Figure 5). Specifically, in the liver tissue, immunohistochemistry demonstrated that RNase1 was mainly produced in the Kupffer and hepatocyte cells (Figure 5A). In gut tissues, RNase1 was not only detected in the intestinal villus and the mucosal layer (Figure 5C), but the muscular layer of the gut tissue (Figure 5D). These results were further confirmed by the immunofluorescence assay (Figure 6). The results of negative controls for the immunohistochemistry assay and immunofluorescence assay were shown in Supplementary Figures S2 and S3, respectively.

### 2.6. Ribonucleolytic Activity of Ma-RNase1

**Figure 7:**
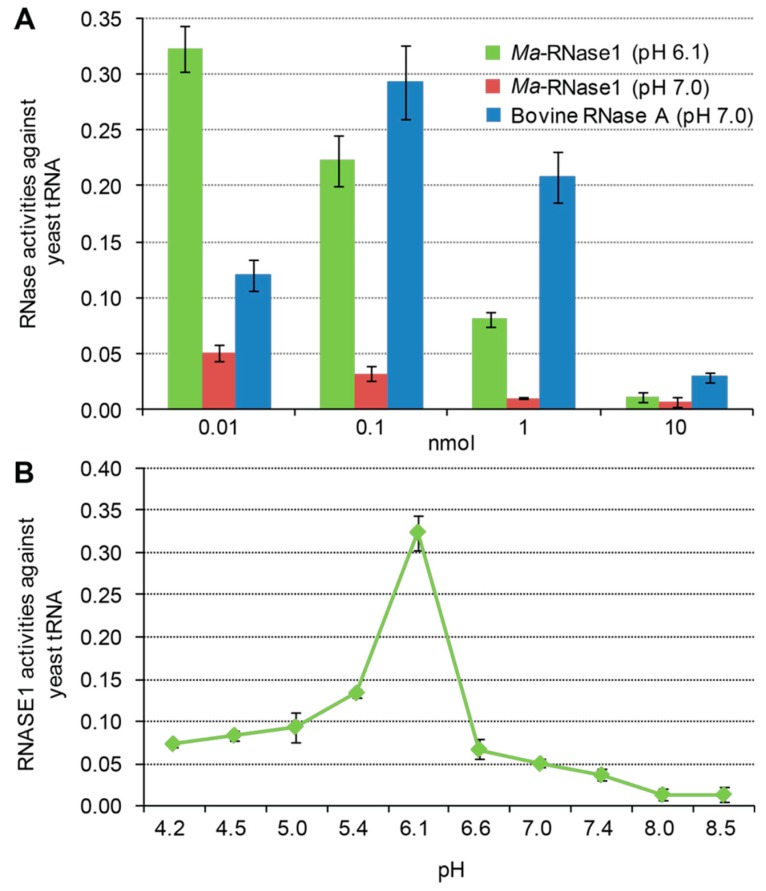
Ribonucleolytic activities of *M. amblycephala* recombinant protein RNase1. Comparison the ribonucleolytic activities of *Ma-*RNase1 and bovine RNase A at different amounts (**A**); RNase activity against yeast tRNA at different pH levels (**B**).

We prepared a recombinant *Ma*-RNase1 protein (Supplementary Figure S4) and determined its ribonucleolytic activity at various amounts and different pHs (Figure 7). As shown in Figure 7A, the catalytic activity of *Ma*-RNase1 was much higher at pH 6.1 than at pH 7.0 and decreased in a dose-dependent manner. Moreover, the activity of *Ma*-RNase1 (pH 6.1) was 2.7-fold at 0.01 nmol, comparable to that of bovine RNase A. Thus, 0.01 nmol *Ma*-RNase1 was as the optimal amount. There were obvious differences in the ribonucleolytic activity of *Ma*-RNase1 at different pH (Figure 7B). The catalytic activity of *Ma*-RNase1 was highest at pH 6.1, which was recognized as its optimal pH (Figure 7B).

## 3. Discussion

RNase1 is one of the most important digestive enzymes and has been widely used for molecular evolutionary and functional biological studies. Previously, RNase1 has been extensively studied in mammals, especially in foregut-fermenting monkeys and bovines, suggesting that the duplication of the *RNase1* gene was an adaptation to their digestive physiology [4,13]. To date, only several RNases from *D. rerio* [19,20,21] and two RNases from *S. salar* [8] have been studied independently in different laboratories.

In this study, we identified single copies of the *RNase1* gene in the genomes of two herbivorous (*M. amblycephala* and *C. idellus*) and three omnivorous fish (*C. carpio*, *D. rerio*, and *O. latipes*). This analysis of the *RNase1* of fish species has added to the growing data on the diversity of *RNase1* genes and their evolution. A multiple-protein-sequence alignment showed that the four cyprinid species RNase1 proteins are most similar to each other, sharing relatively low sequence identity with the mammalian RNase1 proteins. The cyprinid fish RNase1 sequences have all the features of the RNase A superfamily (Figure 1): they are encoded in a single exon, have a signal peptide with the “CKXXNTF” signature motif, and have two critically positioned histidine residues and a lysine residue. However, they differ from mammalian RNases in the number of conserved cysteines: mammalian RNase1 proteins have eight conserved cysteines [22], whereas most nonmammalian RNase1 proteins, including those of fishes, have only six, which is very similar to the human angiogenin proteins (RNase5) [23]. Previous study demonstrated that all vertebrate RNases evolved from angiogenic fish Rnases [24]. With the selection they underwent during evolution, these proteins evolved other diverse bioactivities, including digestive activity, bactericidal activities, and angiogenic activity [6,20]. Therefore, it is not surprising that the *D. rerio* and *S. salar* RNase1 proteins have angiogenic and bactericidal activities [6,8].

Our qPCR analysis showed that the *Ma-RNase1* gene is weakly expressed at 120 and 144 hpf (Figure 4) but no expression was detected in the early developmental stages, which is similar to that in *D. rerio* [19]. *Ma-RNase1* was only expressed in the liver and heart of one-year-old fish. However, in the two-year-old fish, it was expressed in most of the tissues tested, except the heart and muscle. The strongest expression of *Ma-RNase1* was detected in its endocrine tissues (liver, gut, spleen, and kidney), the organs involved in immune and digestion functions. This is consistent with the alternative name for RNase1, “secretory RNase”. The expression profiles of *RNase1* in different fish species and different animals are diverse. High *RNase1* expression in different tissues might indicate multiple functions. For instance, its high expression in the gut may regulate the gut microbiota [19]; in the thymus, it may be involved in immune functions [8]; and in the pancreas, it may be an adaptation to ruminant-like digestion [11,25]. Therefore, the specific expression profiles of the *Ma-RNase1* gene indicate its involvement in multiple functions.

It has been reported that nearly all fish RNases have low ribonucleolytic activity, but powerful bactericidal activity [7,8,19]. Notably, the ribonucleolytic activities of some RNases in the human [26], chicken [27], and fish [7] are not necessary for their bactericidal activities, and the two biological activities are independent. In the present study, we found that *Ma*-RNase1 of the herbivorous fish *M. amblycephala* has strong ribonucleolytic activity at its optimal reaction pH (pH 6.1), similar to that of bovine pancreatic RNase (RNase A) at pH 7.4. Importantly, the optimal reaction pH of *Ma*-RNase1 is similar to that in the intestinal microenvironment of *M. amblycephala* (pH 5.8 ± 0.5). This indicates that the *Ma*-RNase1 protein has undergone selection during evolution and suggests that its strong ribonucleolytic activity is an adaptation to its herbivorous diet. Similarly, in mammals, the duplicated RNase1β, driven by selection in Asian and African leaf monkeys, have their highest ribonucleolytic activity at their optimal pHs (6.3–6.7), matching the pH (6–7) of the colobine small intestine, to maximize food digestion [4,13].

In conclusion, in this study, we performed sequence alignment and phylogenetic analyses of the RNase1 proteins of fishes and mammals, examined the expression profile of *Ma-RNase1* in the early developmental stages and different tissues of one-year-old and two-year-old *M. amblycephala*, and demonstrated the potent ribonucleolytic activity of a recombinant *Ma*-RNase1 protein. These data provide a clear and comprehensive picture of the expression patterns, evolution and the adaptive diversification of a herbivorous fish RNase1.

## 4. Materials and Methods

### 4.1. Sample Collection

All the experimental procedures involving fish were performed in accordance with the guidelines of National Institute of Health Guide for the Care and Use of Laboratory Animals and approved by the Research Ethics Committee, Huazhong Agricultural University, Wuhan, China (HZAUMO2015-0015, Approval data: 16 July 2015). *M. amblycephala* and their fertilized eggs were obtained from the Freshwater Fish Genetics Breeding Center of Huazhong Agricultural University (Wuhan, China). After the specimens anesthetized with MS-222 and sterilized with 75% alcohol, eight different tissues were immediately collected from the one-year-old and two-year-old (adult) *M. amblycephala*: heart, liver, spleen, kidney, brain, muscle, gut, and testis. Samples were also collected from seven different early developmental embryonic stages (hour post-fertilization (hpf)): 0 (fertilized egg), 12 (late gastrula stage), 27 (heart appearance), 40 (hatching), 72 (gill circulation), 120 (air bladder formation), and 144 (intestine appearance). All samples were immediately frozen in liquid nitrogen and stored at −80 °C.

### 4.2. Identification of RNase1 Genes

The coding sequence of *Ma-RNase1* was obtained from our previous transcriptomic data for *M. amblycephala* (GenBank accession numbers: SRX731259 and SRA045792). Possible unigenes annotated as “RNase1” were identified with BLAST homology search in the GenBank database, and conformed to the key features of the known *RNase1* genes. The full-length sequence of *Ma-RNase1* was obtained from the genome data of *M. amblycephala.* We also identified the *RNase1* genes in the genomes of *D. rerio*, *C. carpio*, *C. idellus*, and *O. latipes*. The previously published *RNase1* gene sequences from the National Center for Biotechnology Information (NCBI) database were as query sequences. These were then aligned against each of the genomic sequences (*D. rerio*, *C. carpio*, *C. idellus*, and *O. latipes*) with TBLASTN (Legacy Blast v2.2.23) [28], with a threshold *E*-value < 1 × 10^−10^. Genewise (v2.2.0) [29] was used to predict the gene structures, which were confirmed with nonredundant (NR) annotation. Finally, we searched the four fish genomes for sequences with similarity to the query sequences and containing the key features of the known *RNase1* genes.

### 4.3. Protein Alignment

The RNase1 protein sequences of *Homo sapiens* (ABF00144.1), *Bos taurus* (NP_861526.1), *Ailuropoda melanoleuca* (AHI58810.1), *Myotis altarium* (AGF41059.1–AGF41061.1), *Rattus norvegicus* (EDL88443.1, NP_001013250.1, NP_001025075.1), *Callosciurus prevostii* (ACV70063.1, ACV70064.1), *Arctonyx collaris* (AHI58780.1–AHI58782.1), *Martes flavigula* (AHI58776.1–AHI58778.1), *Colobus guereza* (ABF60825.1–ABF60827.1), and *Pygathrix nemaeus* (AF449642_1 and AF449643_1) were obtained from the NCBI database (www.ncbi.nlm.nih.gov). The *RNase1* gene sequences of *S. salar* and *Oncorhynchus mykiss* were obtained from the study of Cho and Zhang [19]. The protein sequences were aligned with the Muscle program [30]. The SignalP 4.1 server was used to predict the signal peptide cleavage sites [31]. The pI and *M*_W_ of the mature proteins were calculated with the online tool Compute pI/*M*_W_ [32].

### 4.4. Structural Predictions

The three-dimensional structures of four cyprinid fishes (*D. rerio*, *C. carpio*, *C. idellus* and *M. amblycephala*) RNase1, *B. taurus* RNase A and *H. sapiens* RNase5 were modeled with the SWISS-MODEL server [33,34]. According to the results with the lowest *E*-value and the highest score of a BLAST search against the Protein Data Bank database (PDB), the template (PDB ID: 2vq9.1A) was chosen to build the four cyprinid fishes models. The sequence identity between the template and *D. rerio*, *C. carpio*, *C. idellus* and *M. amblycephala* RNase1 was 95%, 61%, 76% and 72%, respectively.

### 4.5. Phylogenetic Analyses

To better understand the evolutionary relationships of the genes encoding RNase1 among the teleosts and mammals, MEGA version 5.1 program [35] was used for evolutionary analyses. Protein sequences were aligned with the Muscle program. The phylogenetic tree was reconstructed using the Neighbor-Joining method with the protein Jones–Taylor–Thornton (JTT) matrix model and 1000 bootstrap replications [36].

### 4.6. Ma-RNase1 Gene Expression Analysis

Total RNA was isolated from each sample with RNAiso Plus (TaKaRa, Dalian, China), according to the manufacturer’s instructions. The quality of the RNA was evaluated with 2% agarose gel electrophoresis and the concentrations were determined with a NanoDrop ND-2000 spectrophotometer (NanoDrop Technology, Wilmington, DE, USA). For qPCR, first-strand cDNA was synthesized from 1 μg of total RNA using a reverse transcriptase kit from TaKaRa Biochemicals (Dalian, China). We amplified the *RNase1* mRNA sequence with the primers: 5′-ACTGGTCCTTTGTGCCTTCT-3′ (forward) and 5′-GGATTGTGTGATGCGTCTGT-3′ (reverse). The primers for the housekeeping gene encoding *β-actin* were 5′-ACCCACACCGTGCCCATCTA-3′ (forward) and 5′-CGGACAATTTCTCTTTCGGCTG-3′ (reverse). SYBR Green PCR Master Mix (TaKaRa, Dalian, China) was used for qPCR and this was performed using a Roter-gene Q (Qiagen, Hilden, Germany). The PCR condition was a cycle of pre-denaturation at 95 °C for 45 s, followed by 40 cycles of amplification at 95 °C for 15 s, 60 °C for 15 s, and 72 °C for 30 s, finally followed by a dissociation curve. The average value per gene was calculated for 3 replicates.

### 4.7. Assessment of Ma-RNase1 Production by Immunostaining

Immunohistochemistry (IHC) assay was performed to evaluate RNase1 expression at the cellular level. Liver, gut and kidney tissues from two-year-old *M. amblycephala* fixed with 4% paraformaldehyde, then embedded in paraffin and sectioned. Following deparaffinization, rehydration and antigen retrieval, slides were blocked using an Avidin/Biotin blocking kit (Vector Laboratories, Inc., Burlingame, CA, USA) followed by serum-free protein block. Then the slides were incubated overnight at 4 °C with monoclonal rabbit RNase1 antibody (prepared in this study) diluted 1:300 in phosphate-buffered saline (PBS). After washing with PBS, the slides were incubated with secondary antibody IgG-HRP (Santa Cruz Biotechnology Inc., Santa Cruz, CA, USA) at room temperature for 1 h and visualized in DAB substrate solution. For immunofluorescence detection, all sections were prepared as outlined above except developing using mounting media with 4′,6-diamidino-2-phenylindole (DAPI). All the slides were examined with an inverted fluorescent microscope (Nikon, Co., Tokyo, Japan) and photographed with DP70 digital camera (Olympus, Tokyo, Japan).

### 4.8. Recombinant Protein Preparation

The signal peptide sequence of *Ma*-RNase1 was predicted with the SignalP 4.1 server. The cDNA fragment encoding RNase1, but lacking the sequence encoding the signal peptide, was amplified with the following primers: 5′-GACACGGATCCGAAAATCTGTACTTCCAAGGT-3′ (forward) and 5′-GTGTCCTCGAGTTATACAATGACACCTTCTTCATA-3′ (reverse). The underlined letters indicate the restriction sites added to allow cloning. The reaction was performed with *Taq* DNA polymerase (TaKaRa). The amplified *Ma-RNase1* fragment was treated with BamHI and XhoI restriction endonucleases and inserted into the bacterial expression vector pET32a (Novagen, Madison, WI, USA). The purified plasmid DNA was verified with sequencing before processing. *Escherichia coli* strain Rosetta (DE3) was transformed with the expression plasmid containing the cDNA encoding the mature peptide region of *Ma-RNase1*. The recombinant protein was isolated, purified, and quantified as previously described [20].

### 4.9. Assays of Ma-RNase1 Activity

The RNase1 activity of the recombinant protein in a standard yeast tRNA substrate assay was determined in 40 mM Na-4-(2-hydroxyethyl)-1-piperazineethanesulfonic acid (Na-HEPES) buffer with pHs ranging from 4.0 to 9.0 at 25 °C [4]. To determine the optimal reaction amount of the tested protein, we added four amounts of purified *Ma*-RNase1 protein (0.01, 0.1, 1, or 10 nmol) to 0.8 mL of Na-HEPES buffer (pH 7.0) with 1.42 nmol of tRNA. For comparison, in another experiment, we added a series of amounts of commercial bovine RNase A (Sigma-Aldrich, St. Louis, MO, USA). We also measured the *Ma*-RNase1 activity of the recombinant protein against yeast tRNA by adding the optimal amount of RNase1 and 1.42 nmol of tRNA to 0.8 mL of 10 buffers with varying pHs (pH 4.0–9.0). The reactions were terminated by the addition of 1 mL of 20 mM freshly prepared lanthanum nitrate (Sigma) with 3% perchloric acid (Sigma), and incubated on ice for 15 min. The mixtures were then centrifuged at 12,000× *g* for 15 min at 4 °C to remove any insoluble tRNA. The amount of solubilized tRNA was calculated from the ultraviolet absorbance at 260 nm, and the catalytic activity of RNase1 was defined as previously described [26].

## Supplementary Materials

Supplementary materials can be found at http://www.mdpi.com/1422-0067/17/5/786/s1.
