# Supplementary material for: Expression Patterns and Functional Novelty of Ribonuclease 1 in Herbivorous Megalobrama amblycephala
Source: Int J Mol Sci. 2016 May 20;17(5):786. doi: 10.3390/ijms17050786 (PMC4881602; doi:10.3390/ijms17050786)
Supplement: Supplementary file 1 [file ijms-17-00786-s001.pdf]

Supplementary Materials: Expression Patterns and Functional Novelty of Ribonuclease 1 in Herbivorous *Megalobrama amblycephala*

Han Liu and Weimin Wang

```
       ggcacttattttcattcattattttcttcattaatctcccaaaacactttatttattca
       actgactgtagttagtgtcttgatatatgatgtattgctcaccaggtc
       ATG GGT ATT CAT CAG TCT ACA GTG ATT GTG CTA CTG GTC CTT TGT
1       M   G   I   H   Q   S   T   V   I   V   L   L   V   L   C
       GCC TTC TTC TCA TTG TCT ATT TAT GGT CAA CCA GCA GAA GTA AGG
16      A   F   F   S   L   S   I   Y   G   Q   P   A   E   V   R
       CGA CGT TAT GAG CAT TTC CTT ACG CAG CAT GTG TAT GGA GCC ATG
31      R   R   Y   E   H   F   L   T   Q   H   V   Y   G   A   M
       ACT GAG CAG AGA TGT GAC AGG GTC ATC CGC GAC AGA CGC ATC ACA
46      T   E   Q   R   C   D   R   V   I   R   D   R   R   I   T
       CAA TCC CAA AAT GGC AAC GAC TGC AAA GAA GTC AAC ACC TTC ATA
61      Q   S   Q   N   G   N   D   C   K   E   V   N   T   F   I
       CAG GCA AAT AGT AAC CAA GTT AGA GCA GTT TGT ACT GGG GCT GGA
76      Q   A   N   S   N   Q   V   R   A   V   C   T   G   A   G
       ACT CGA CTC CAT GAA AAC AGA GAT TTG TTT ATT AGC GAA TAT CCA
91      T   R   L   H   E   N   R   D   L   F   I   S   E   Y   P
       TTT CCT GTG GTT ACA TGT ACA TTA AGA AGA GGG GAA AGA CGG CCA
106     F   P   V   V   T   C   T   L   R   R   G   E   R   R   P
       AAC TGT AAT TAC AGA GGT CAC TTG TCC ACT CGC AAA ATT GTT GTG
121     N   C   N   Y   R   G   H   L   S   T   R   K   I   V   V
       GCA TGT GCA GAC AAG TGG CCA GTA CAC TAT GAA GAA GGT GTC ATT
136     A   C   A   D   K   W   P   V   H   Y   E   E   G   V   I
       GTAtaaatgtcaaactgcatagatgttttatcatgacccaataaataatcaaacacacaa
151     V  *
       tcagataattctgttagatgcaatgatgttcatgagt
```

Figure S1. Nucleotide sequence of the *M. amblycephala RNase1* gene. 5′-and 3′-untranslated regions are shown in lower case. The start codon (ATG) and asterisk indicated the stop codon (taa) is boxed. The putative polyadenylation site (aataaa) is underlined. The conceptually translated protein sequence is given below the DNA sequence, with the signal peptide boldfaced. Numbers in the left showed the numbers of *MA*-RNase1 amino acids.

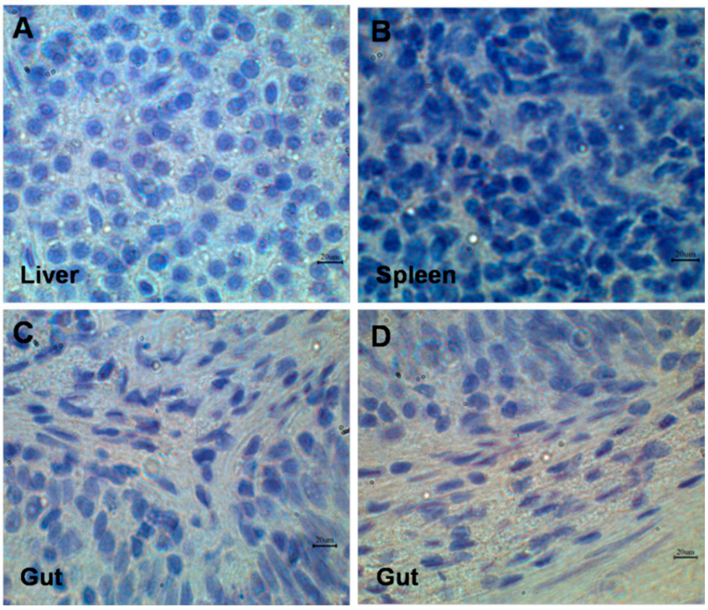

Figure S2. Negative control for immunohistochemstry assay (serum of rabbit before being immunized replace primary antibody). Liver (**A**), spleen (**B**) and gut (**C**,**D**).

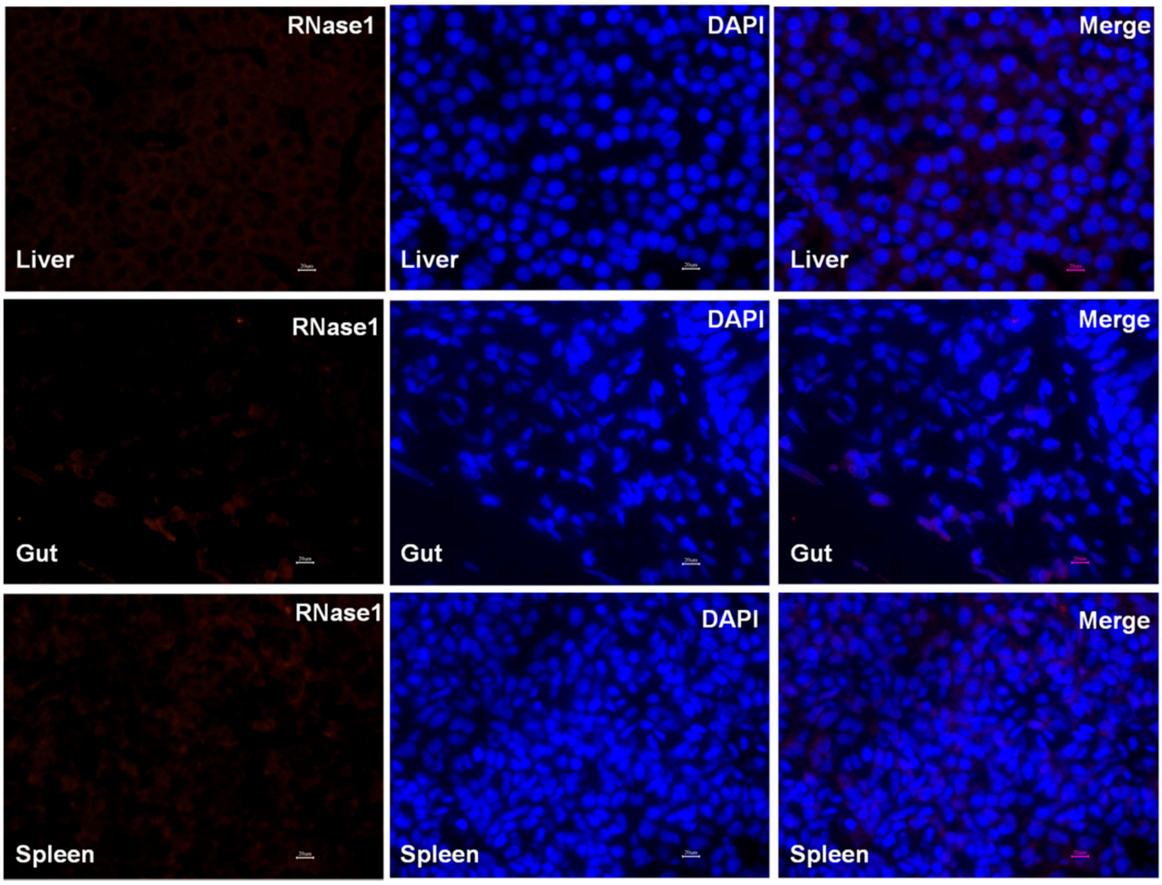

Figure S3. Negative control for immunofluorescence assay (serum of rabbit before being immunized replace primary antibody).

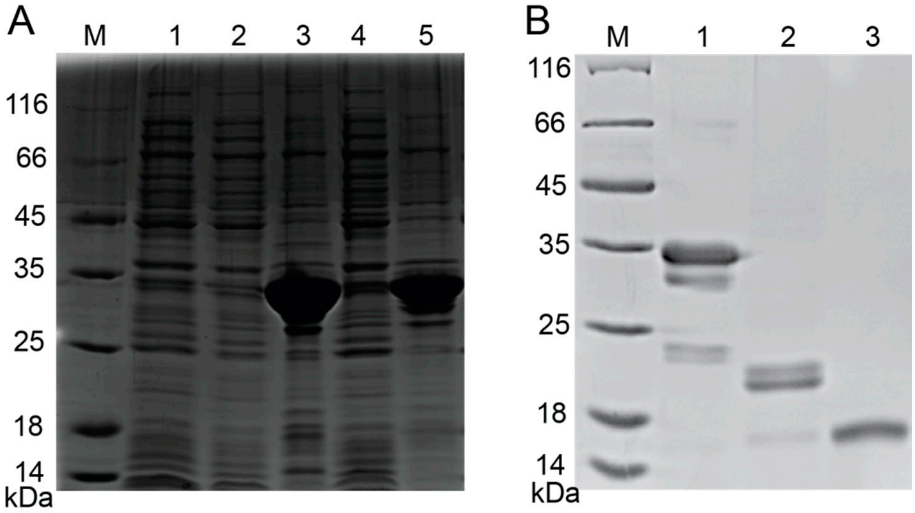

Figure S4. SDS-PAGE analysis of recombinant fusion *Ma*-RNase1 protein. (**A**) Optimization of the induction conditions of recombinant *Ma*-RNase1expression. M, protein molecular weight marker; Lane 1, total protein before induction; Lanes 2 and 4, supernatants of cell lysis after induction at 20 and 37 °C, respectively; Lanes 3 and 5, total proteins after induction at 20 and 37 °C, respectively; (**B**) Purification of target protein. M, protein molecular weight marker; Lane 1, *Ma*-RNase1 fusion protein; Lane 2, TEV-digested *Ma*-RNase1 fusion protein; Lane 3, purified target protein.
